# Supplementary figures and images for: Transition from infectivity and immune escape to pure escape as an evolutionary strategy during the COVID-19 pandemic
Source: bioRxiv. 2026 Feb 27:2026.02.26.706090. Preprint. [Version 1] doi: 10.64898/2026.02.26.706090 (PMC13160004; doi:10.64898/2026.02.26.706090)

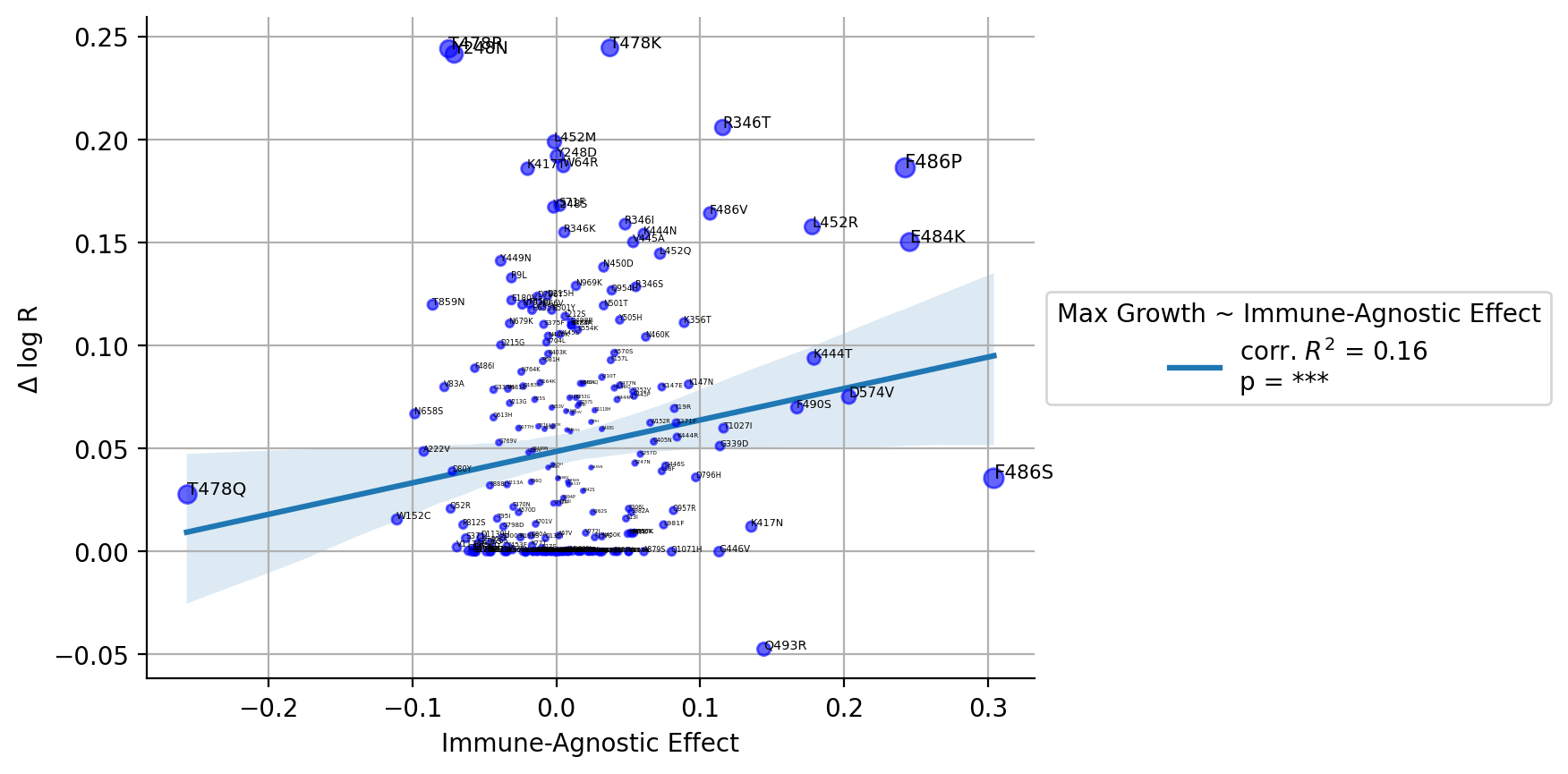

Supplement: Supplement 1 [file media-1.zip › Supplemental Figures/PyR0-Escape.png]

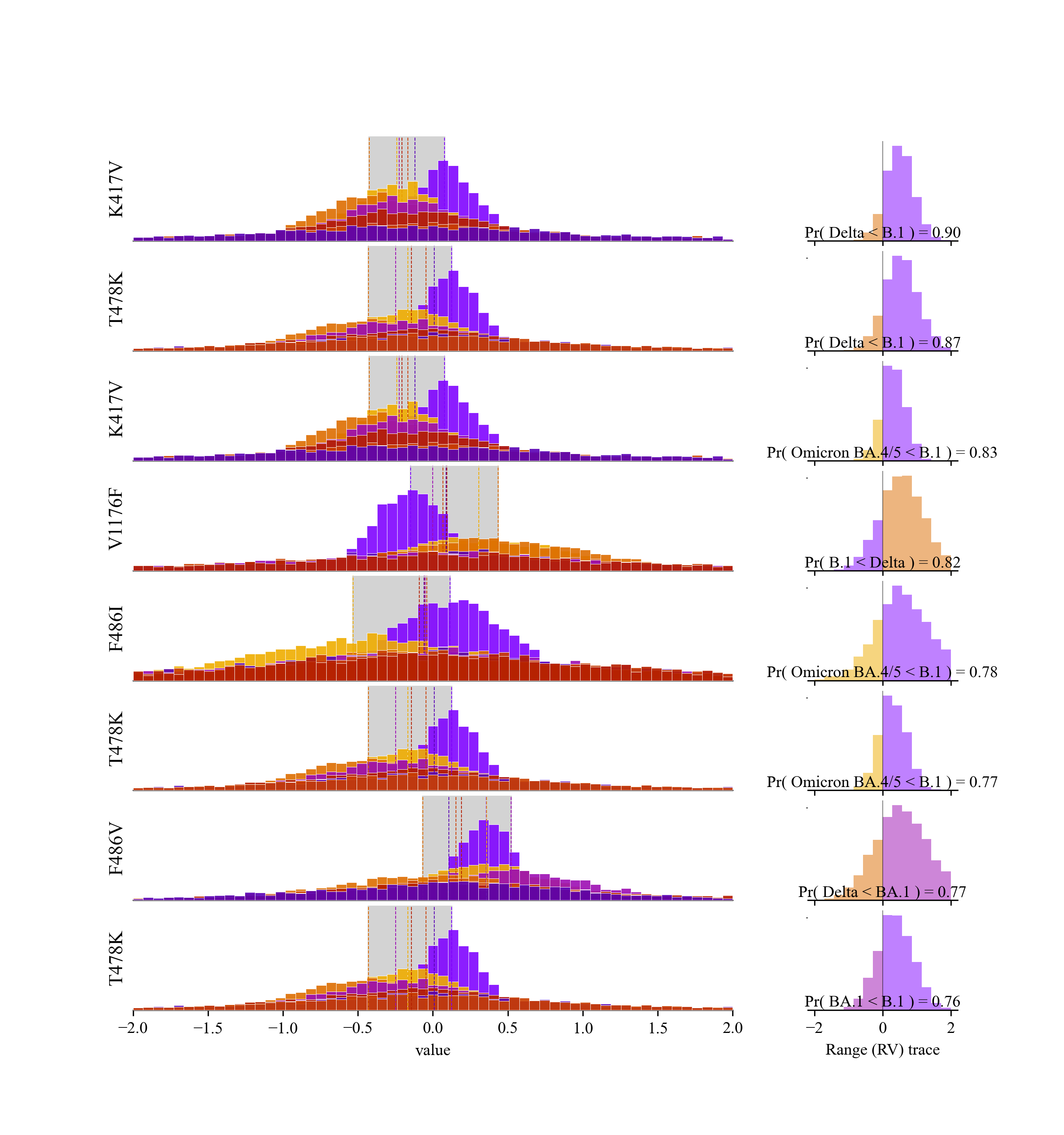

Supplement: Supplement 1 [file media-1.zip › Supplemental Figures/S6.png]

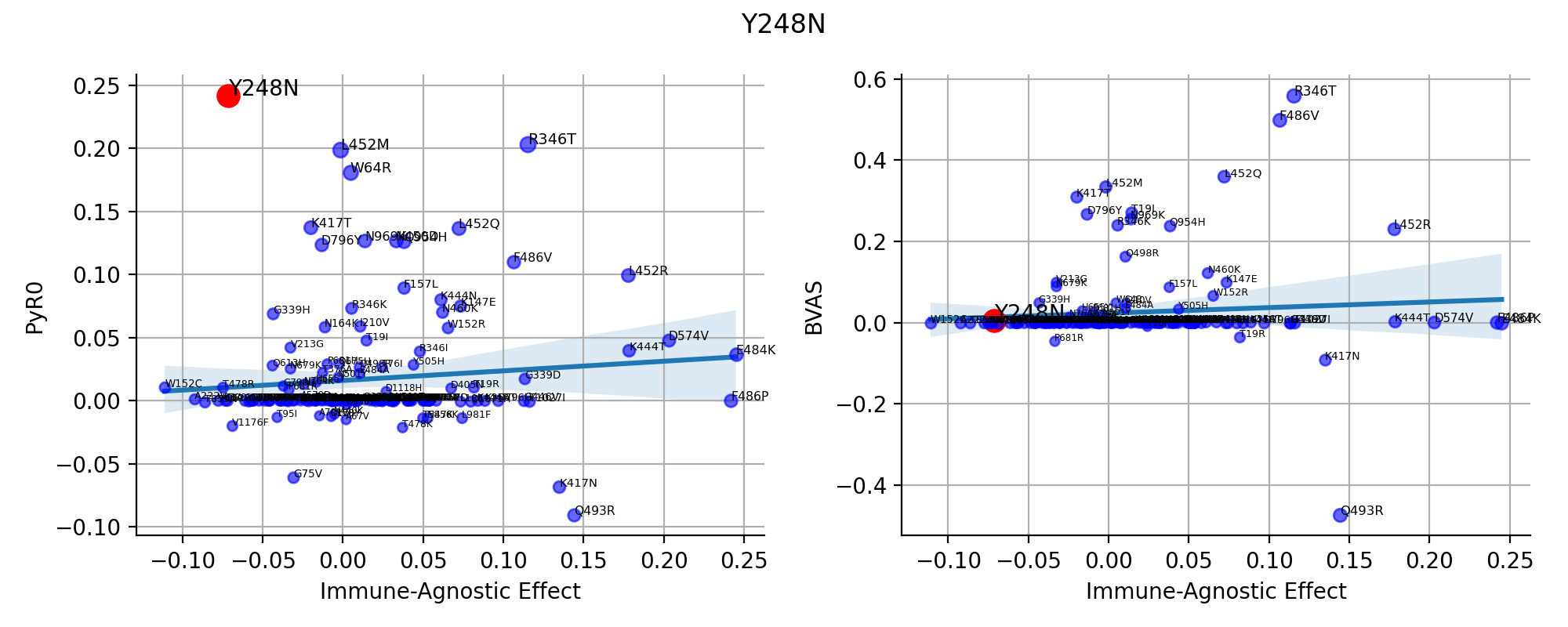

Supplement: Supplement 1 [file media-1.zip › Supplemental Figures/S13.png]

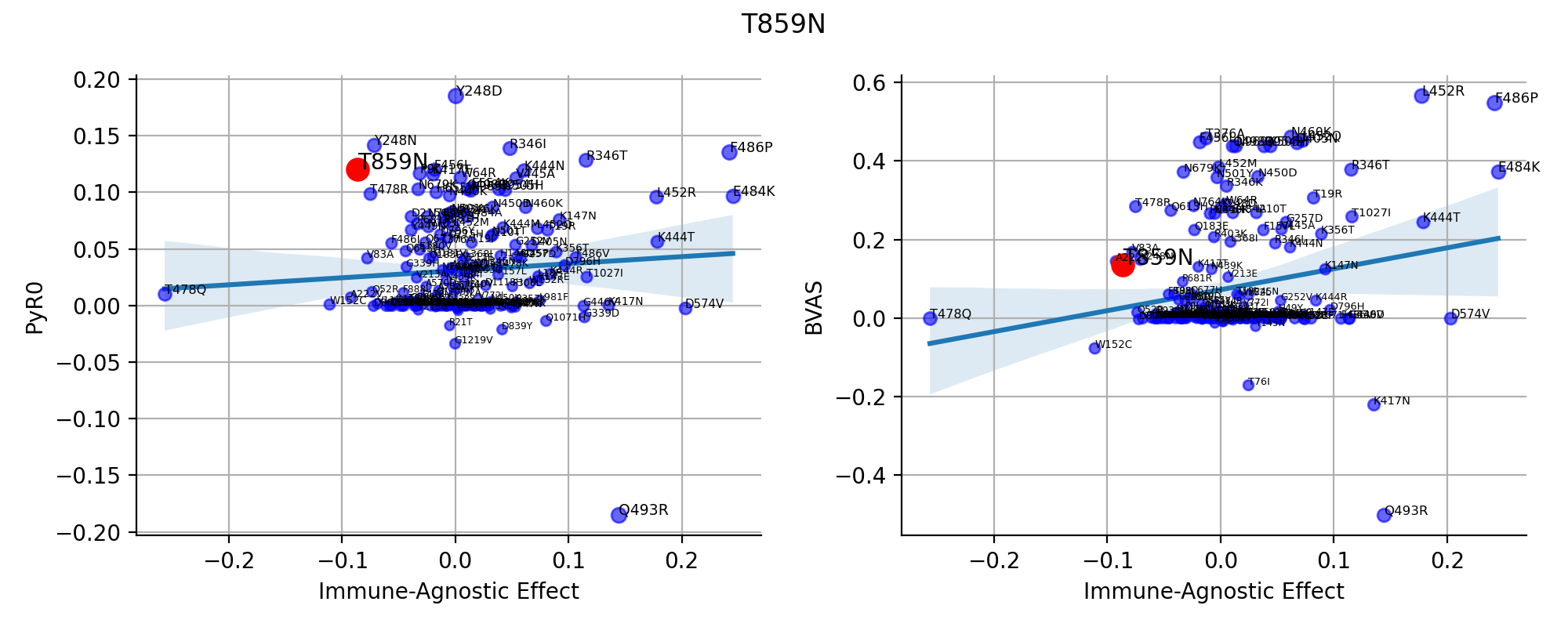

Supplement: Supplement 1 [file media-1.zip › Supplemental Figures/S12.png]

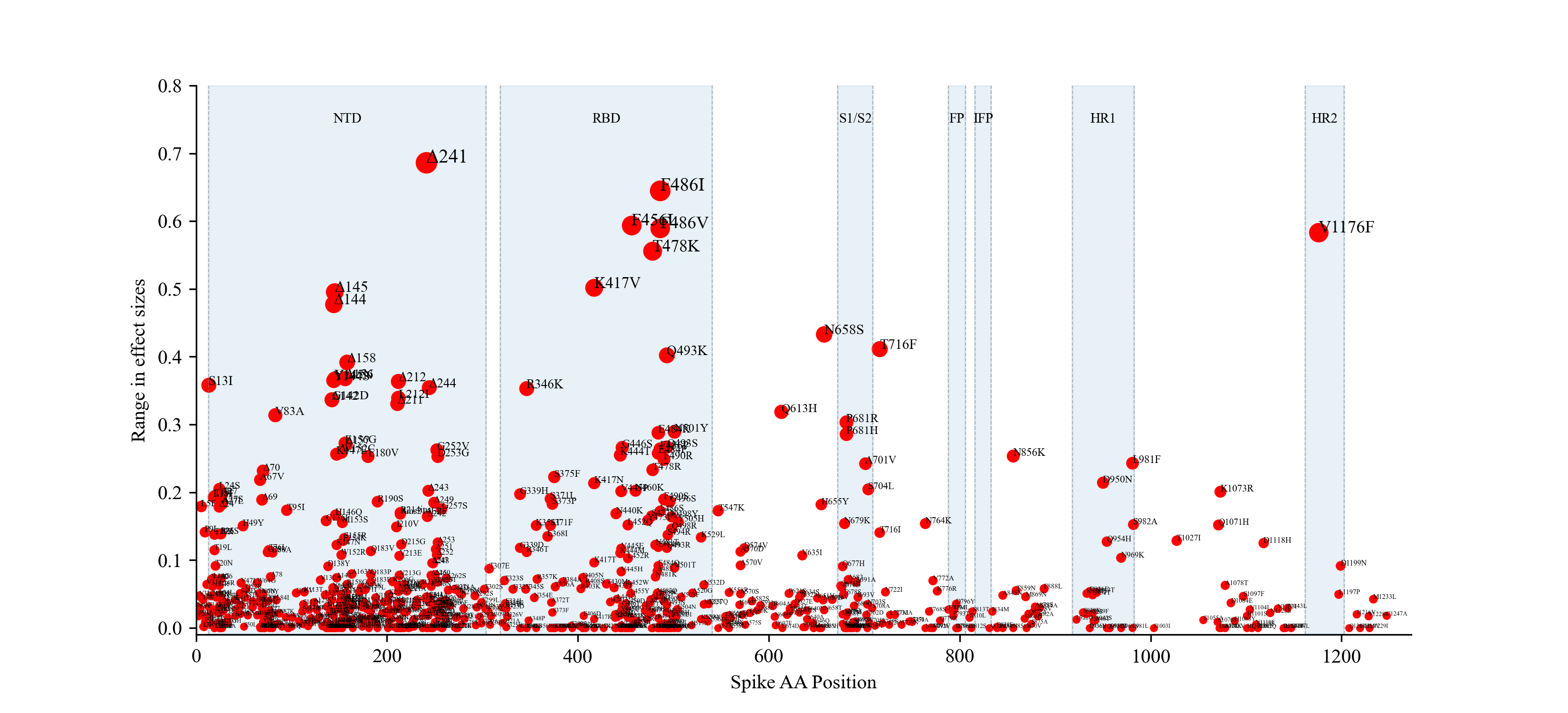

Supplement: Supplement 1 [file media-1.zip › Supplemental Figures/S7.png]

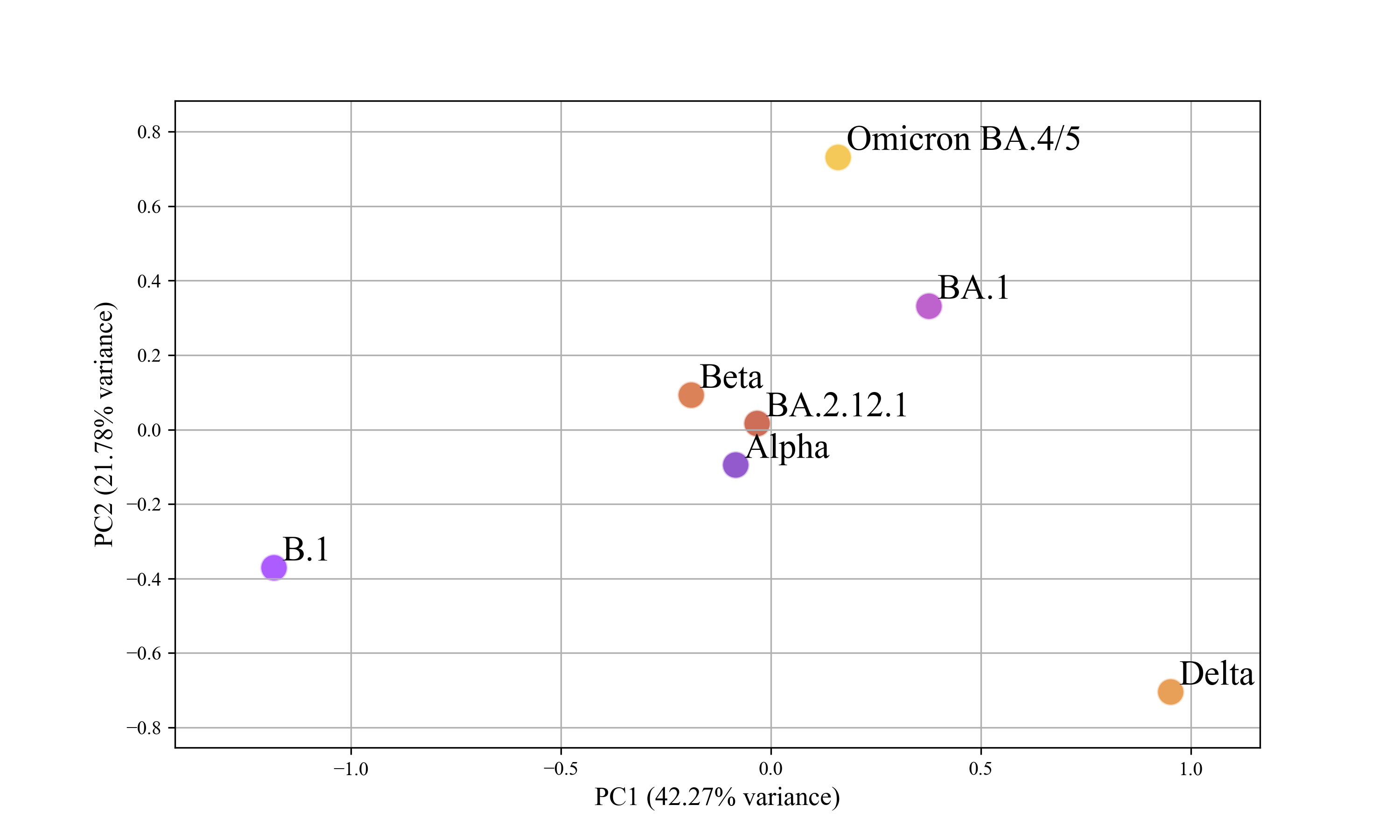

Supplement: Supplement 1 [file media-1.zip › Supplemental Figures/S5.png]

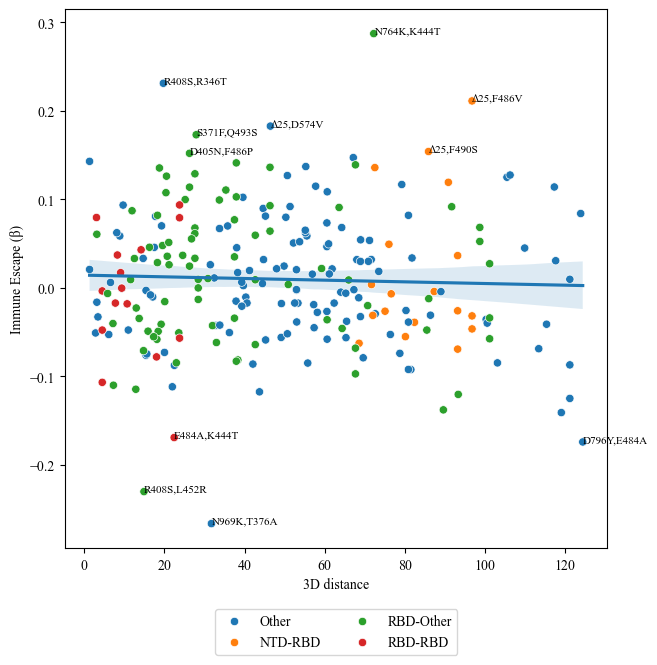

Supplement: Supplement 1 [file media-1.zip › Supplemental Figures/S10.png]

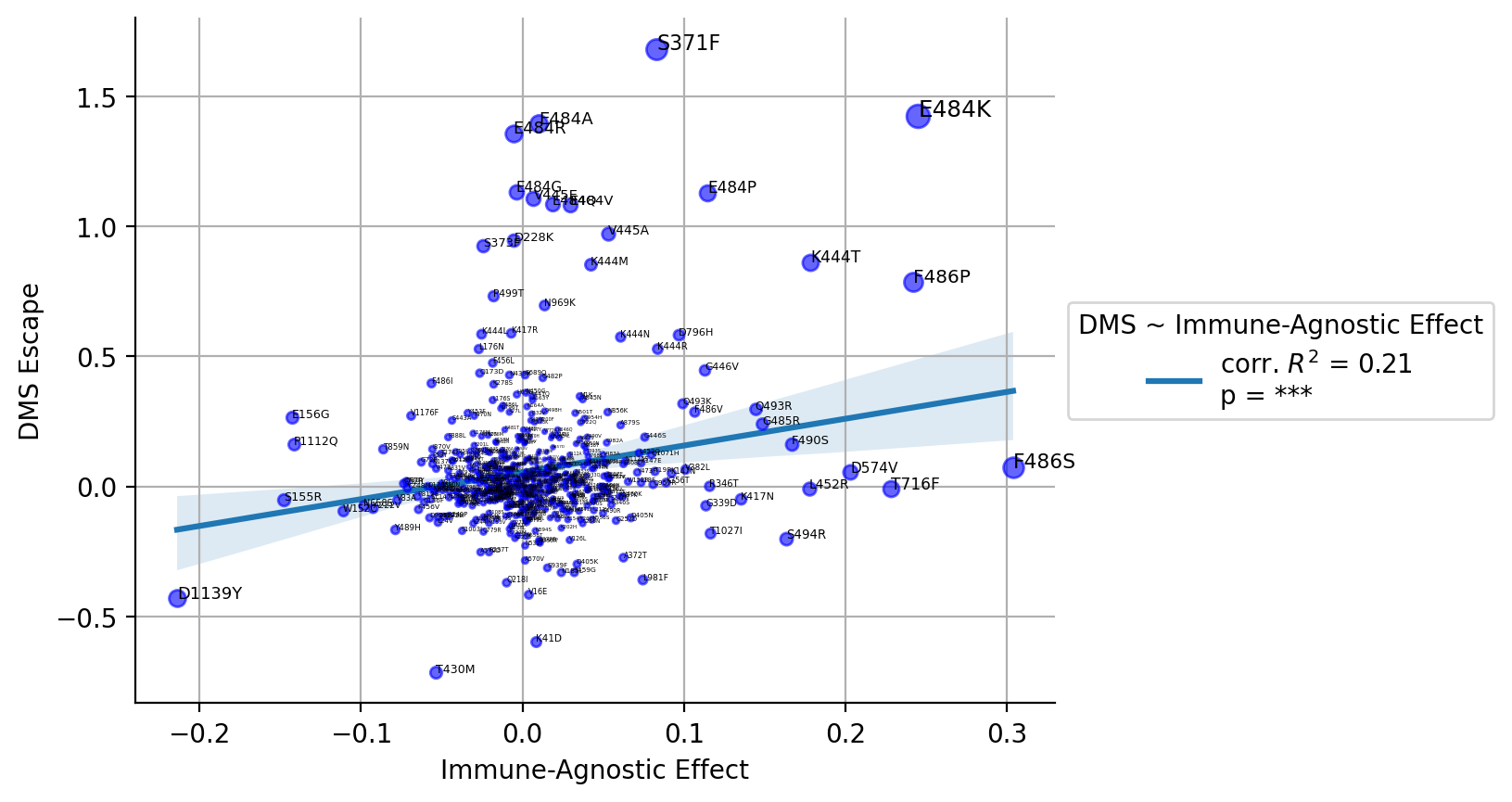

Supplement: Supplement 1 [file media-1.zip › Supplemental Figures/DMS-Escape.png]

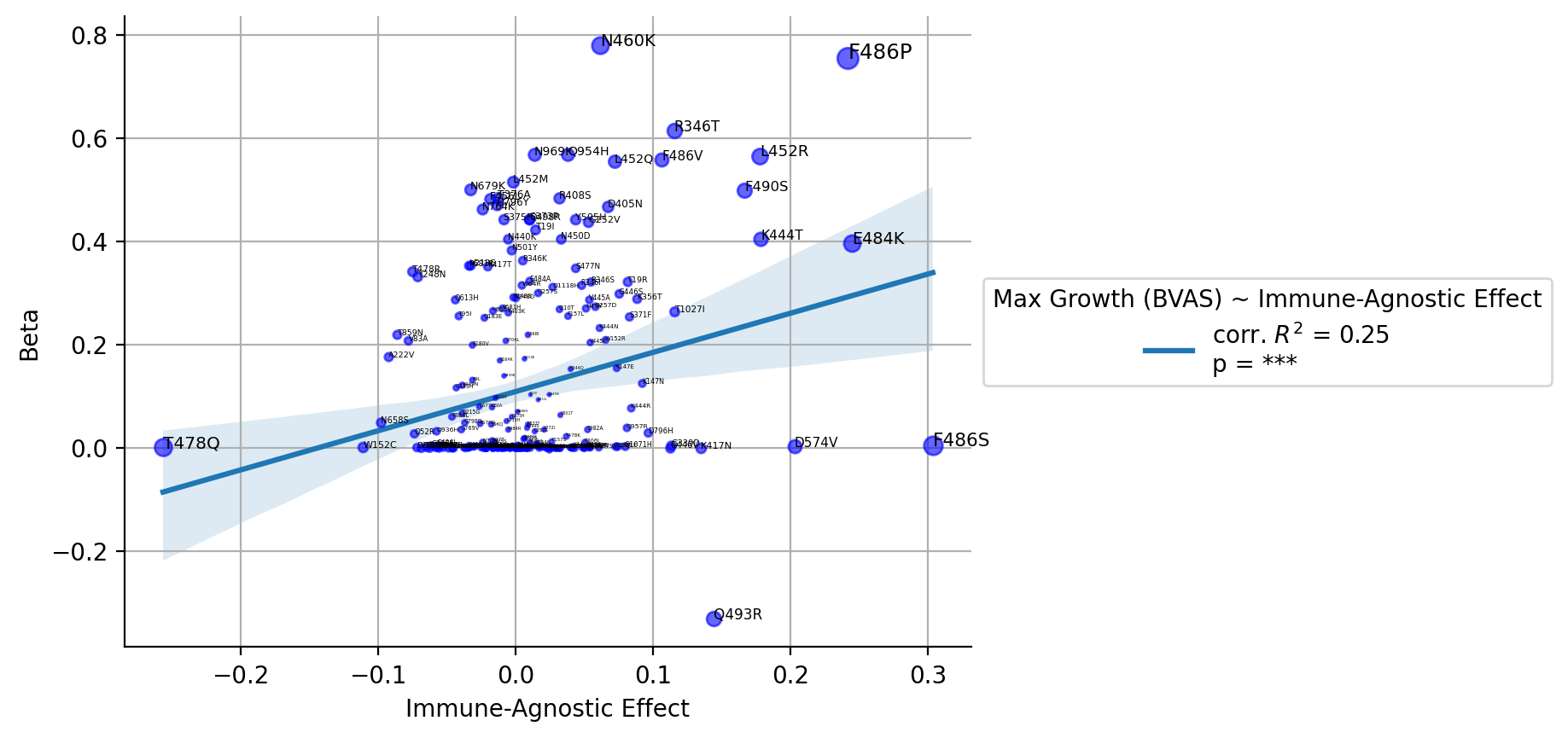

Supplement: Supplement 1 [file media-1.zip › Supplemental Figures/S11.png]

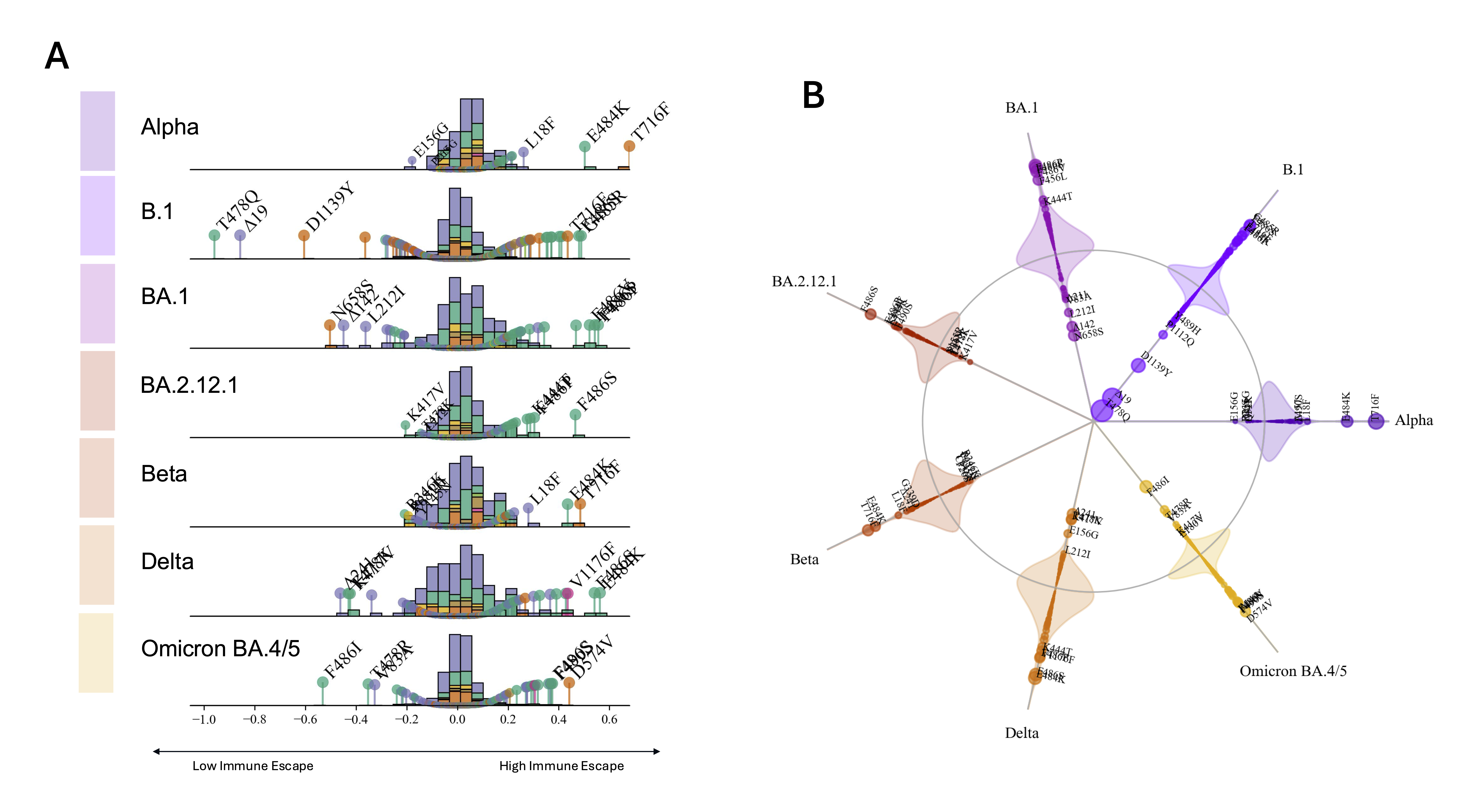

Supplement: Supplement 1 [file media-1.zip › Supplemental Figures/S4.png]

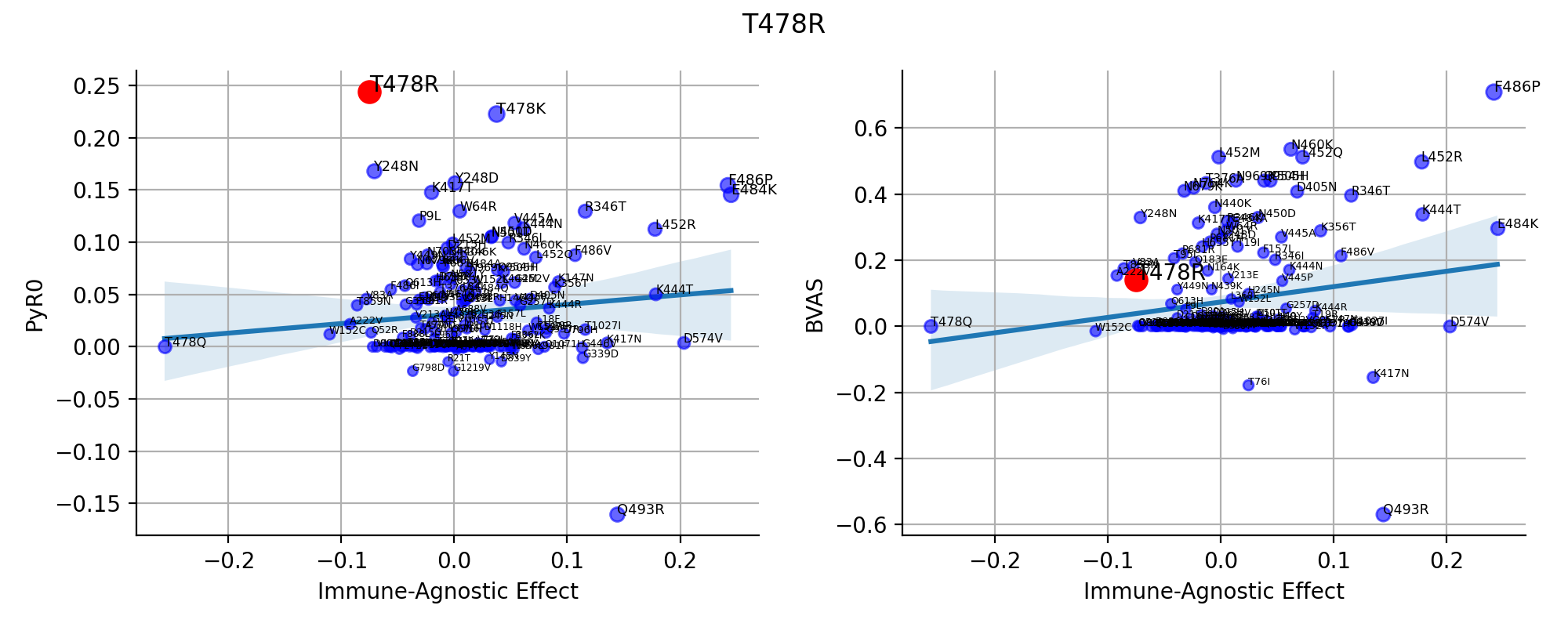

Supplement: Supplement 1 [file media-1.zip › Supplemental Figures/S15.png]

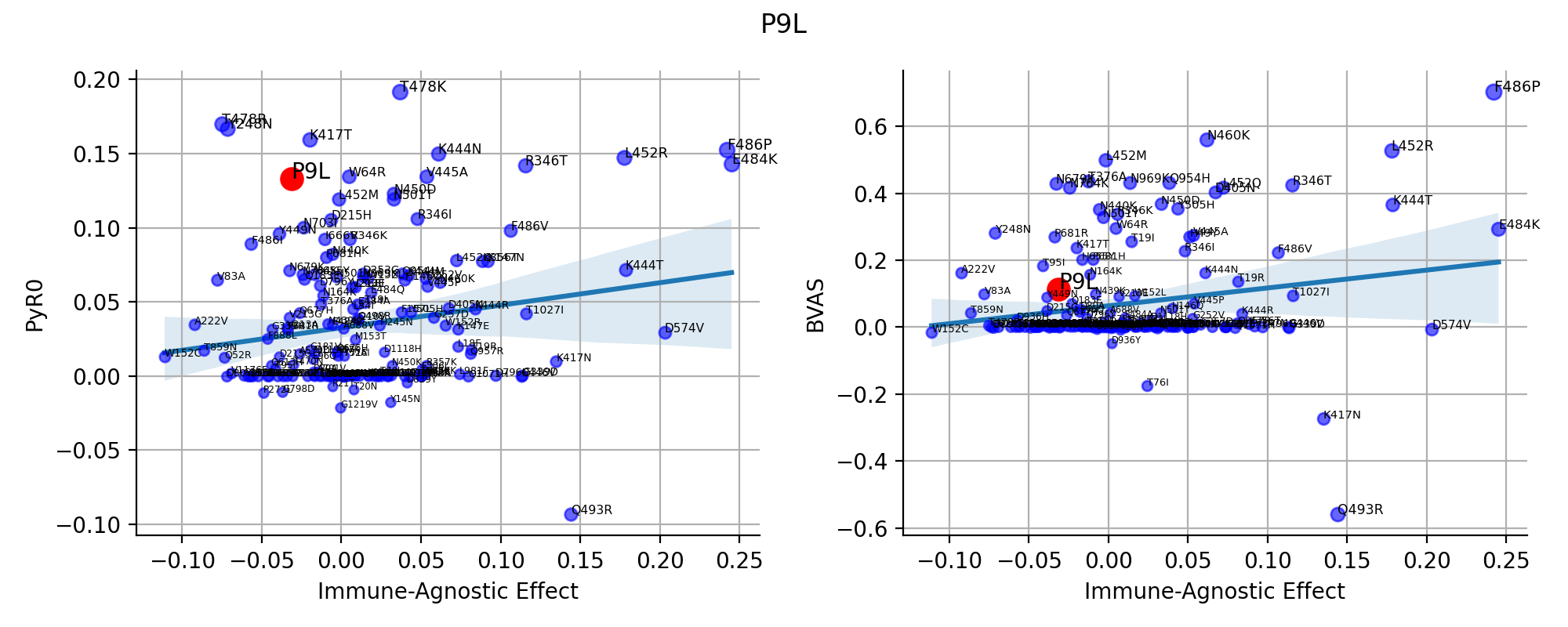

Supplement: Supplement 1 [file media-1.zip › Supplemental Figures/S14.png]

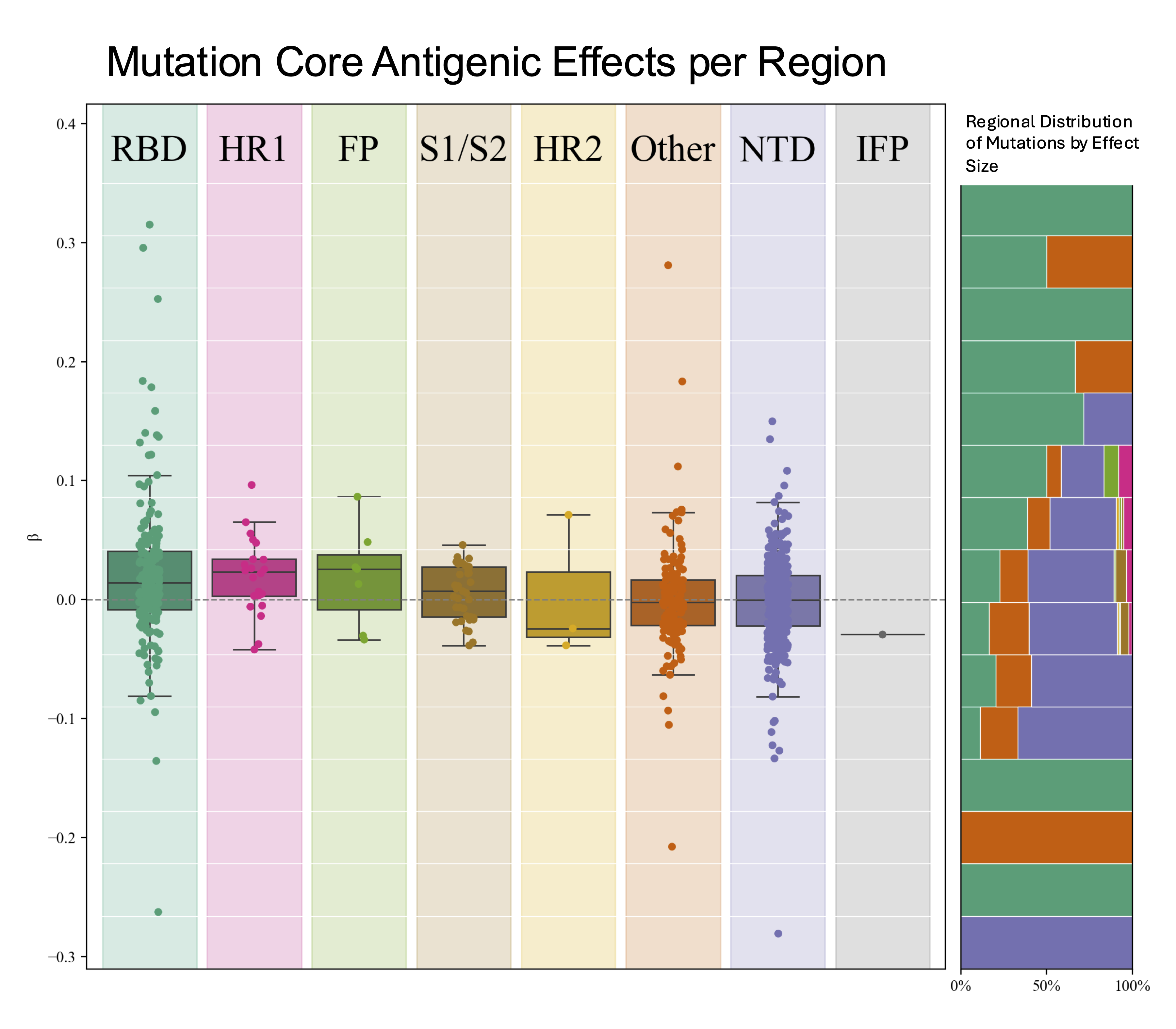

Supplement: Supplement 1 [file media-1.zip › Supplemental Figures/S1.png]

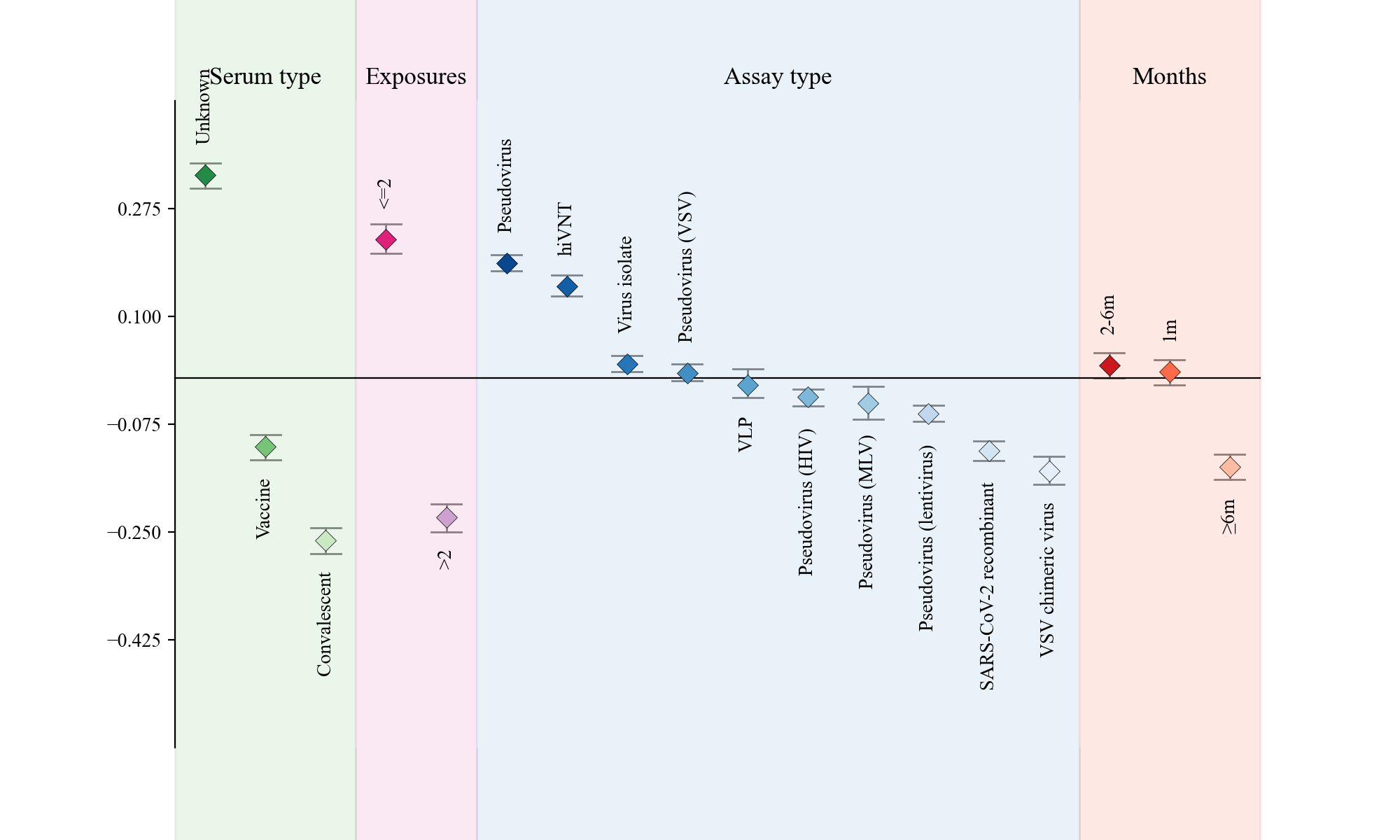

Supplement: Supplement 1 [file media-1.zip › Supplemental Figures/S3.png]

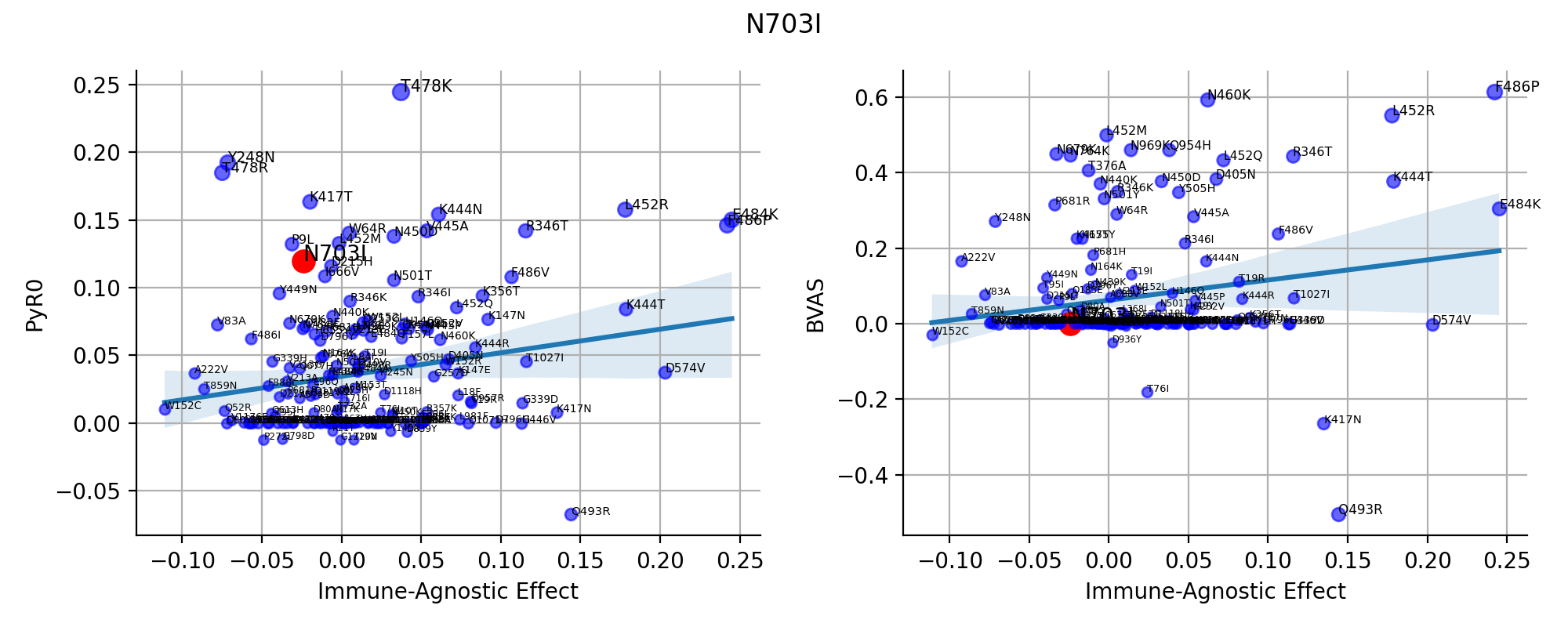

Supplement: Supplement 1 [file media-1.zip › Supplemental Figures/S16.png]

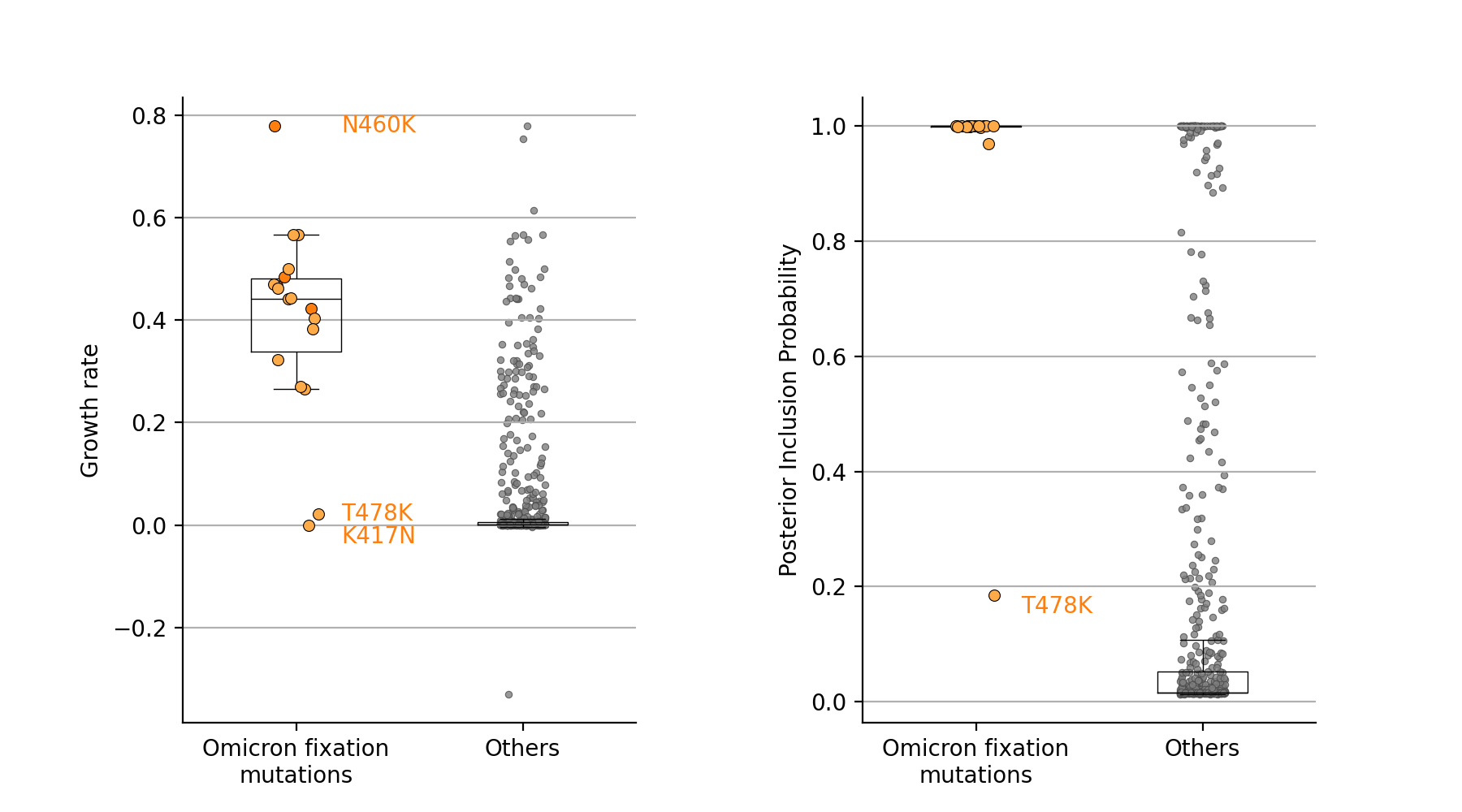

Supplement: Supplement 1 [file media-1.zip › Supplemental Figures/S17.png]

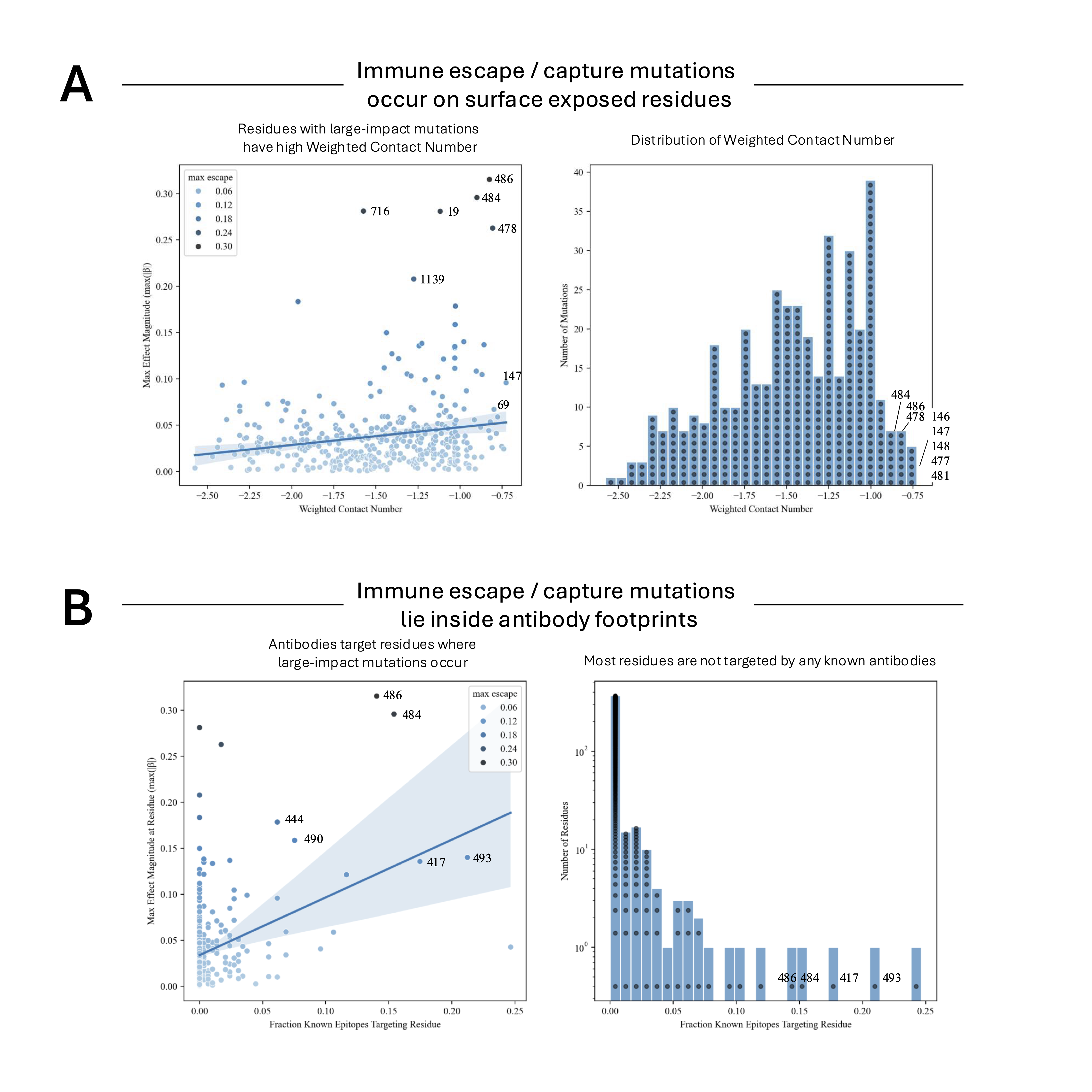

Supplement: Supplement 1 [file media-1.zip › Supplemental Figures/S2.png]

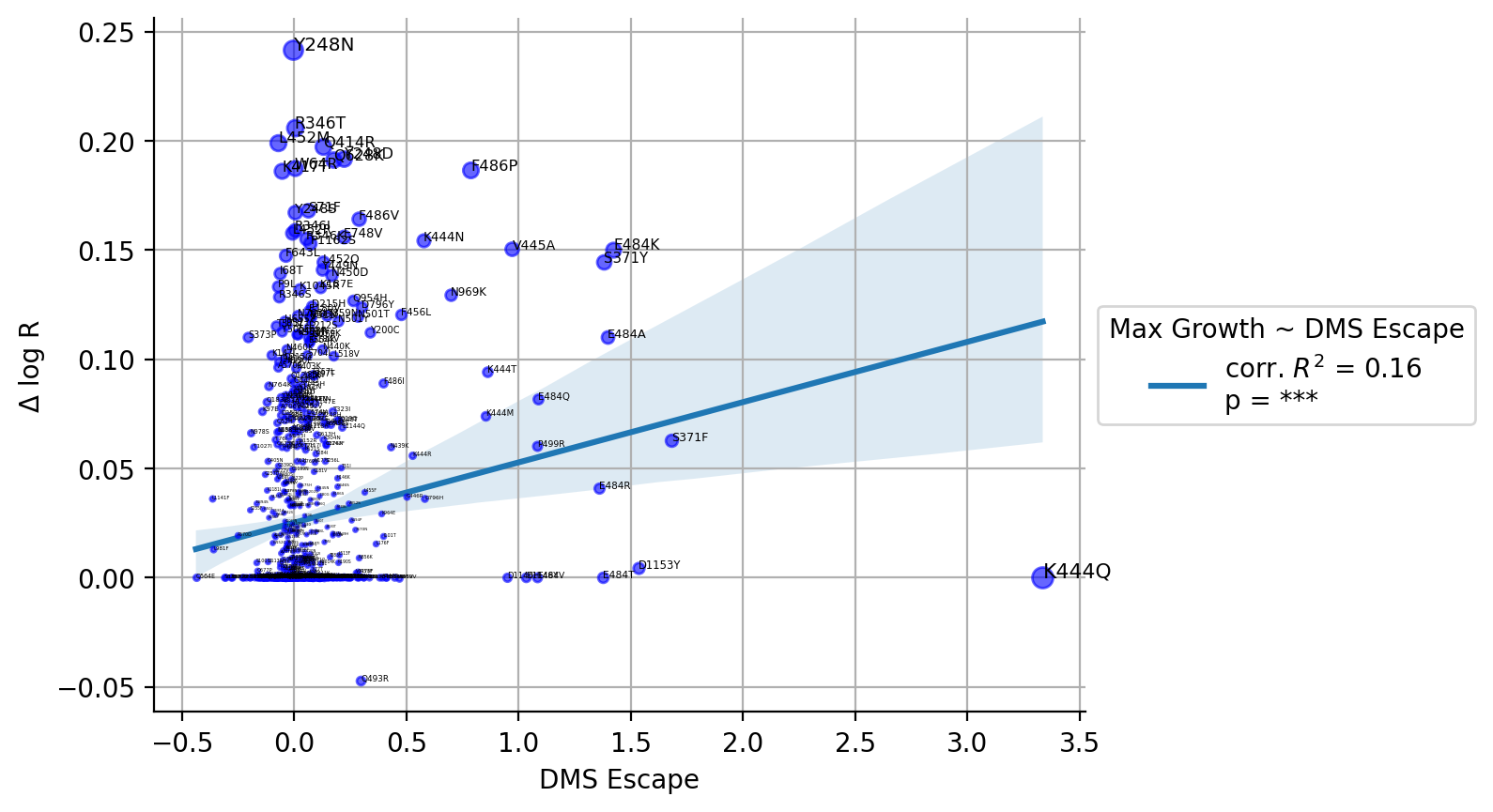

Supplement: Supplement 1 [file media-1.zip › Supplemental Figures/PyR0-DMS.png]

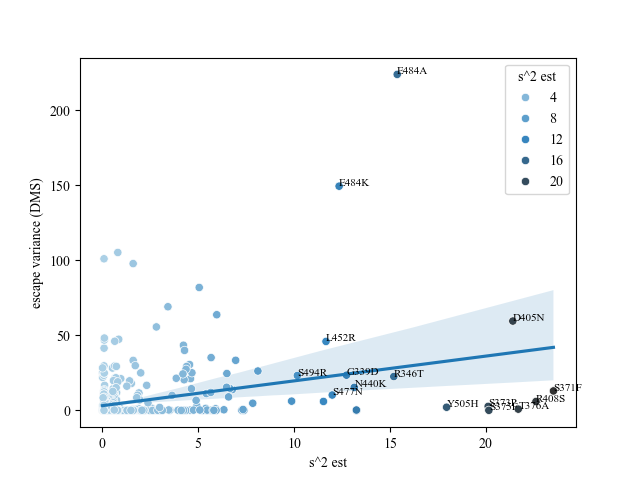

Supplement: Supplement 1 [file media-1.zip › Supplemental Figures/S9.png]

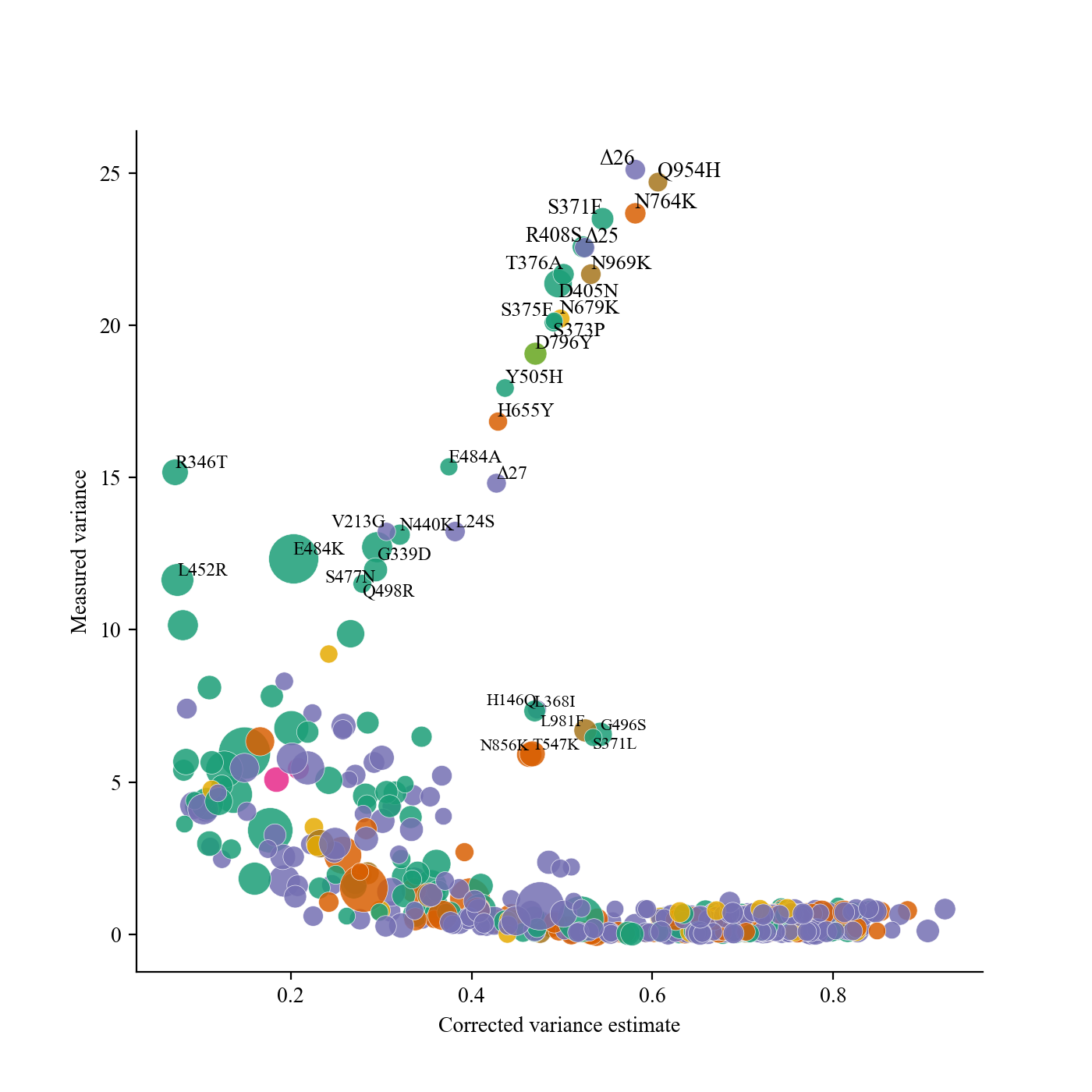

Supplement: Supplement 1 [file media-1.zip › Supplemental Figures/S8.png]
