# Supplementary material for: Transition from infectivity and immune escape to pure escape as an evolutionary strategy during the COVID-19 pandemic
Source: bioRxiv. 2026 Feb 27:2026.02.26.706090. Preprint. [Version 1] doi: 10.64898/2026.02.26.706090 (PMC13160004; doi:10.64898/2026.02.26.706090)
Supplement: Supplement 2 — Table S1:Serum Pool and Core Antigenic Effects for each mutation Table S2: Variance decomposition of each mutation Table S3: EPI_SET [file media-2.zip › Supplemental Files/S3.pdf]

SUPPLEMENTAL TABLE

Data Availability

GISAID Identifier: EPI_SET_250808ux
DOI: https://doi.org/10.55876/gis8.250808ux

All genome sequences and associated metadata in this dataset are published in GISAID's EpiCoV database. To view the contributors of each individual sequence with details such as accession number, Virus name, Collection date, Originating Lab and Submitting Lab and the list of Authors, visit EPI_SET_250808ux

Data Snapshot

EPI_SET_250808ux is composed of 17,243,458 individual genome sequences.
The collection dates range from 2019-12-05 to 2025-06-15;
Data were collected in 222 countries and territories.
